# Impact Load Behavior between Different Charge and Lifter in a Laboratory-Scale Mill

**DOI:** 10.3390/ma10080882

**Published:** 2017-07-31

**Authors:** Zixin Yin, Yuxing Peng, Zhencai Zhu, Zhangfa Yu, Tongqing Li

**Affiliations:** 1 School of Mechatronic Engineering, China University of Mining & Technology, Xuzhou, 221116 China; yinzixincumt@126.com (Z.Y.); zhuzhencai@vip.163.com (Z.Z.); litongqingcumt@163.com (T.L.); 2 Jiangsu Key Laboratory of Mine Mechanical and Electrical Equipment, China University of Mining & Technology, Xuzhou 221116, China; 3 Luoyang Mining Machinery Engineering Design Institute Co., Ltd., Luoyang 471039, China; citichic006@126.com; 4 State Key Laboratory of Mining Heavy Equipment, CITIC Heavy Industries Co., Ltd., Luoyang 471039, China

**Keywords:** DEM simulation, load behavior, impact force, power draw, ball mill

## Abstract

The impact behavior between the charge and lifter has significant effect to address the mill processing, and is affected by various factors including mill speed, mill filling, lifter height and media shape. To investigate the multi-body impact load behavior, a series of experiments and Discrete Element Method (DEM) simulations were performed on a laboratory-scale mill, in order to improve the grinding efficiency and prolong the life of the lifter. DEM simulation hitherto has been extensively applied as a leading tool to describe diverse issues in granular processes. The research results shown as follows: The semi-empirical power draw of Bond model in this paper does not apply very satisfactorily for the ball mills, while the power draw determined by DEM simulation show a good approximation for the measured power draw. Besides, the impact force on the lifter was affected by mill speed, grinding media filling, lifter height and iron ore particle. The maximum percent of the impact force between 600 and 1400 N is at 70–80% of critical speed. The impact force can be only above 1400 N at the grinding media filling of 20%, and the maximum percent of impact force between 200 and 1400 N is obtained at the grinding media filling of 20%. The percent of impact force ranging from 0 to 200 N decreases with the increase of lifter height. However, this perfect will increase above 200 N. The impact force will decrease when the iron ore particles are added. Additionally, for the 80% of critical speed, the measured power draw has a maximum value. Increasing the grinding media filling increases the power draw and increasing the lifter height does not lead to any variation in power draw.

## 1. Introduction

**Figure 1:**
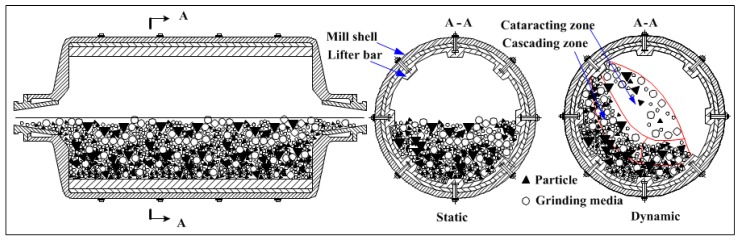
Schematic diagram of ball mill.

A ball mill (Figure 1) is the key piece of equipment for secondary grinding after crushing and is suitable for grinding all types of ores and other materials. Ball mill are used in the mining, cement, chemical and agricultural industries, particularly tumbling ball mills [1,2,3,4]. The comminution process is dependent on the rotation of the mill to lift the grinding media for reducing particle size, including abrasion, crushing and impacting. Due to the impact load behavior between charge and lifter direct impact of milling efficiency, the efficient operation of mill working parameters is a critical issue.

For ball mills, it is dependent on the rotation of the mill shell for lifting the grinding media to impact the particle, and the mill shell, grinding media, particle and lifter are contents of the grinding environment [5,6]. The grinding efficiency is affected by operating variables, such as the charge, lifter and controlling parameter. Thus far, many direct and indirect methods have been employed to measure the impact load behavior, such as X-rays [7], vibration acceleration [8,9,10], acoustic emissions [11], strain [12], force sensor [13], conductivity [14] and inductivity [15]. 

The charge consists of particle and grinding media. Charge parameters include powder filling, grinding media filling and charge size. The particles are grinding material, which are used to further comminute to obtain a suitable size distribution. The grinding media is used for impact-breakage and abrasion. Moreover, the size and filling of grinding media has a strict influence on the milling process [16,17,18,19,20]. Rezaeizadeh et al. [21] studied the effect of the grinding media filling on impact load, concluding that the impact load on the lifters increases as the filling level decreases. Rajrajamani [22] investigated the effects of grinding media size and filling level on the impact spectra, noting that impact spectra changes with filling level and cannot be analyzed accurately. Additionally, as the steel ball size increases, the impact frequency decreases, but the impact forces increases. The purpose of the lifter is lifting charge and preventing slip, and lifter parameters, such as size, shape and number of lifters have a significant effect on load behavior [23,24]. Rezaeizadeh et al. [21] mounted a quartz force sensor to measure the impact load on the lifter. The results showed that increasing the lifter height and number of lifters results in larger impact forces. Djordjevic et al. [25] used the Particle Flow Code 3D (PFC3D) to model the effect of lifter number, height and mill speed on net power draw, concluding that the net power draw decreases as the lifter height increases. Controlling parameters, such as mill speed and mill filling, have been investigated to determine a suitable parameter combination that requires higher grinding efficiency. Hence, many researchers have studied the effect of controlling parameter on milling efficiency [26,27,28]. For mill speed, impact load increases with mill rotation speed, but higher speeds result in the wearing of lifters and increased energy consumption. For mill filling, a minor filling level results in economic cost increases, and an excessive filling level result in mill does not break the particle.

Currently, there are two methods to measure the impact force on the lifter. Method 1 changes in accordance to the drop height of charge in the mill, but transforms into measuring the single charge impact on the lifter material in free fall [29,30,31,32,33,34,35,36]. Method 2 consists of installing a sensor on the mill shell to measure the impact signal of the charge for replacing the impact force of the lifter [20,22,37]. Based on recent research for improving milling efficiency by selecting an appropriate combination of lifter design, mill speed and filling, little is known about a direct measure of the impact force of the lifter and the impact breakage behavior between different shapes of grinding media and lifters. Thus, it is significant to study the impact behavior between charge and lifter.

To investigate the multi-body impact breakage behavior, we designed a drop ball tester and laboratory-scale mill to measure the power draw and impact force on the lifter. In this paper, we primarily studied the effects of mill speed (50–100% of critical speed), grinding media filling (5–30%), lifter height (10–20 mm) and grinding media shape (square, ball and cylinder) on impact breakage behavior. This improved understanding of the impact process in the ball mills and also improved mill grinding efficiency.

## 2. Power Draw Model of Discrete Element Method

The DEM is a numerical method for simulating and analyzing the bulk performance of granular materials and many geomaterials such as coal, ores and rocks. This method was designed to simulate dynamic systems of particles where each element is considered to be an independent and non-deformable entity that interacts with other particles by the laws of contact mechanics and moves following Newton’s second law. This method applies solid-rigid mechanics at the particle level, and the discrete elements are considered to be rigid, non-deformable elements. The constitutive model or behavior of the material is established at the contact areas between particles.

This method has proven to be a useful tool in the milling industry. According to DEM theory, the power draws of ball mills can be calculated as follows:

(1) $$P_{DEM}=m_{c}\times g\times x\times w$$

where *P_DEM_* is the power in watt; *m_c_* is the effective mass of charge; *g* is the acceleration of gravity; *x* is the distance between the center of the mill and the center of the gravity of load; and *w* is the rotational angular velocity of ball mill. 

In the last century, excluding the DEM model of mill power prediction, many models applied to predict the power draw of tumbling mills were very similar [38]. Bond [39] applied the data of industrial mills and laboratory test results to obtain a semi-empirical power draw model; the prediction equation is as follows:

(2) $$P_{Bond}=\beta \times m_{c}\times \left[4.879\times D^{0.3}\times \left(3.2-3\times \varphi \right)\times \psi \times \left(1-\frac{0.1}{2^{9}-104}\right)+1.102\times \frac{d-12.5\times D}{50.8}\right]$$

where *P_Bond_*is the power in watt; *β* is the mill coefficient (*β* = 1.08); *D* is mill diameter; *φ* is mill filling; *Ψ* is mill speed; and *d* is particle diameter in meter. 

## 3. Materials and Methods

### 3.1. Experimental Setup

#### 3.1.1. Drop Ball Experiments

In the ball mill, iron ore particles are broken by the grinding media acting on high-speed crushing and grinding. Reduction of the size of iron ore particles is caused by a cumulative process of single breakage behavior with a longer period of time. To understand the iron ore particle size reduction process, the single breakage behavior must be investigated.

**Figure 2:**
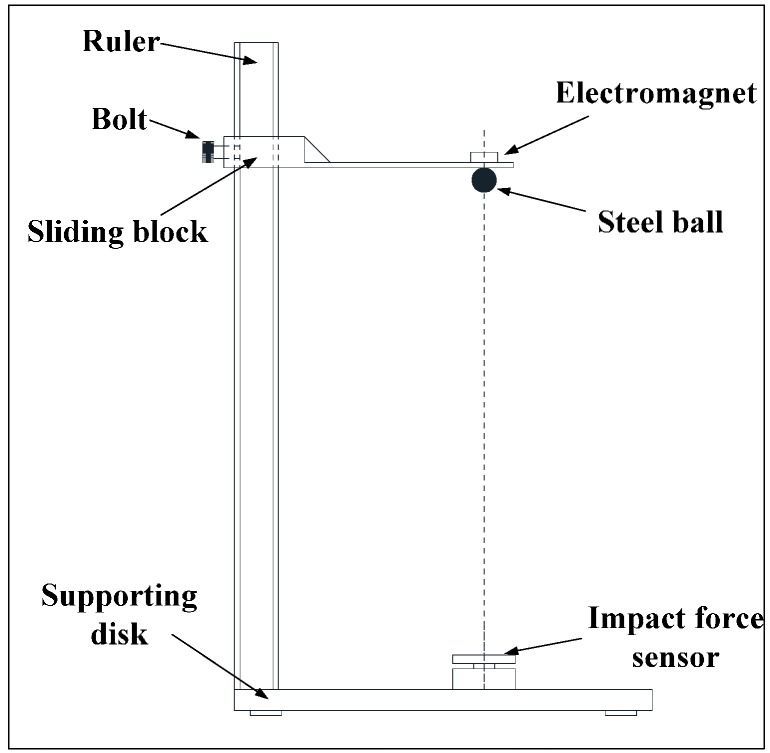
Drop ball tester.

To measure the single breakage behavior, a drop ball tester was designed, as shown in Figure 2. The drop ball tester mainly consists of an impact-force sensor, data acquisition equipment and a supporting disk. Firstly, we moved the sliding block to adjust the drop ball at the right height. Secondly, we dropped the ball to impact the sensor. Lastly, we collected the data of impact force using data acquisition equipment. From the tester, we changed the steel ball size (∅20, ∅30, ∅40 mm) and the drop height (50, 120, 220, 320, 420 and 520 mm) and then obtained a better understanding of the relation between impact force and time.

#### 3.1.2. Impact Breakage Experiments

**Figure 3:**
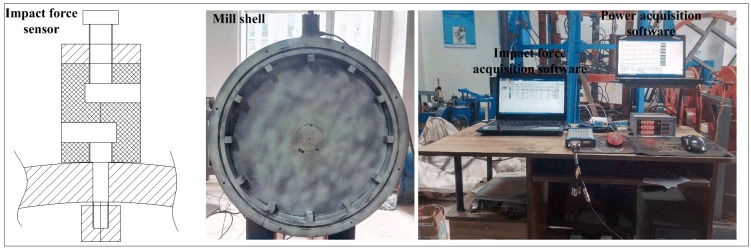
Laboratory-scale mill and data acquiring system.

To carefully collect the load behavior on the lifter, a laboratory-scale mill was designed. Figure 3 shows the laboratory-scale mill and data acquisition system, and the relative facilities are as follows: (i) A mill shell with 520 mm in diameter and 40 mm in length and equipped with 12 equally spaced and different height rows of rectangle lifters; (ii) A 3.7-kW three-phase motor with a rated speed of 1480 rpm; (iii) An inverter was used to control the motor velocity; (iv) A torque sensor with a range of 500 Nm was mounted between the motor and mill shell to measure mill velocity, torque and power by the M400 data acquisition software; (v) A slip ring was mounted on the shaft to prevent winding and supply power; (vi) An impact force sensor mounted through the mill lifter walls was employed to measure the impact force on the lifter, with a sensor range of 300 kg, and the output signal through slip ring to the DH5981 data acquisition equipment with a sampling rate of 20 kHz.

In Figure 3, the experiments were performed in dry conditions, the mill shell with one transparent end (semicircle perspex) and a high-speed digital camera is used to record the charge trajectory, and the milling process was run 3 min and the data was collected for 10 s. The summary of the experimental conditions is given in the Table 1 below.

### 3.2. Materials

Table 2 shows the material properties of the grinding media, iron ore particle and lifter. The grinding media was forged in steel forging with round, square and cylinder shapes with equal mass from Jinan, China. The raw iron ore particle from the Xuzhou iron ore factory was a magnetite (Fe_2_O_3_ 67.46%) pellet. Since grinding fineness has a great effect on iron ore floatation, appropriate grinding fineness can not only guarantee good monomer dissociation, but also help to avoid excessive grinding [23,40]; we choose iron ore feed with 2–2.8 mm. Iron ore particles were broken into suitable sizes with an industrial jaw crusher, then the products were sieved for 10 min by a vibrating screen to acquire mono-sized particles (2–2.8 mm). The lifter was designed with different heights, and the material was the same as the mill shell.

Table 3 shows the input material parameter in the DEM simulations.

## 4. Results and Discussion

### 4.1. Effect of Steel Ball Size and Drop Height on Impact Force

**Figure 4:**
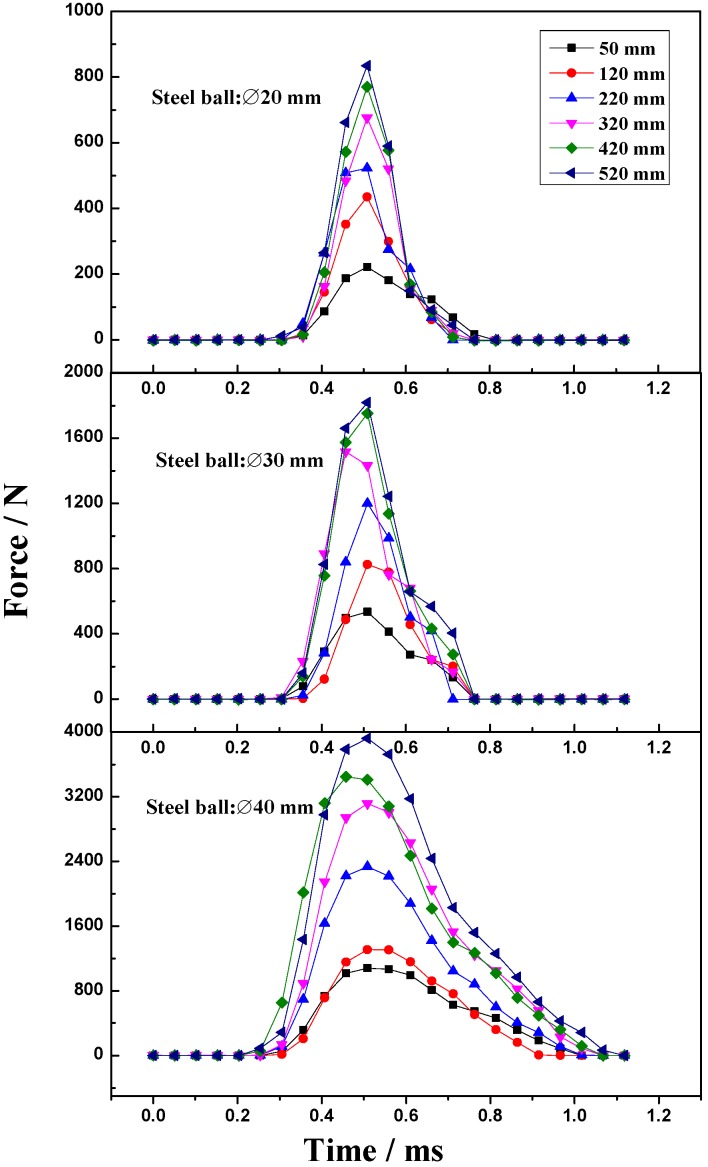
Impact force-time analysis curve for a steel ball with diameters of 20, 30 and 40 mm dropped from different heights.

The impact force plotted against the time in this research is characterized using DH5981 data acquisition equipment collected from the drop ball test performed on lifter. The drop test is performed using different drop height and using three different ball sizes, 20, 30 and 40 mm. The results presented in Figure 4 show that the impact force shows a strong sensitive to the ball size and drop height. The variation in impact force increases up to a maximum and decreases rapidly, and the impact duration time is approximately 1 millisecond. Additionally, the maximum magnitude of impact force increases with the increase of drop height for the given ball size for the given drop height.

Experimental results indicate that each breakage event depends on the steel ball size and drop height, and increasing the steel ball size and drop height increases the gravitational potential energy. Hence, increasing the steel ball size and drop height can increase the impact force in ball mills.

### 4.2. Effect of Mill Speed on Impact Load Behavior

#### 4.2.1. Steel Ball Trajectories of DEM Simulations and Experiments

**Figure 5:**
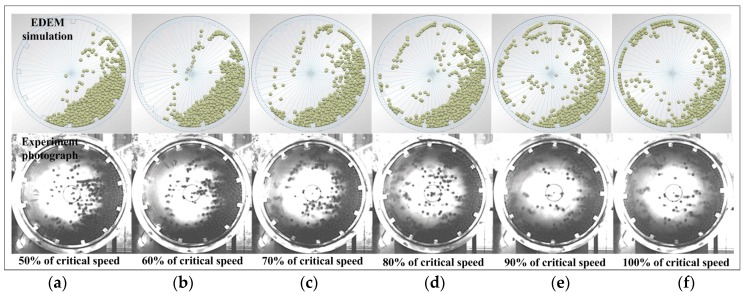
Photographs of the impact load behavior with a grinding media filling of 20% by volume at 50–100% of critical speed: (**a**) 50%; (**b**) 60%; (**c**) 70%; (**d**) 80%; (**e**) 90%; and (**f**) 100%.

In the experiment, the mill fitted with a transparent end, and a high-speed digital camera is used to record steel ball trajectories. Figure 5 shows the steel ball trajectories of DEM simulations and experiments with different mill speeds, the mill speed changed from 50% to 100%, a lifter size of 40 × 20 × 20 mm, and a grinding media filling of 20% by volume. The photograph results are shown the grinding information inside a mill, which includes the steel ball trajectories and the particular charge position. From the photographs of the DEM simulation and experiment, more steel balls are projected into flight to impact the mill shell as the rotating velocity of the mill increases. The steel ball behaviors are highly consistent in the DEM simulation and experiment. At 50% and 60% critical speed, the steel ball move from the bottom of the shell to the shoulder position begin to slide downward across the free surface, and much higher part of steel balls follow a cascading motion. At 70% and 80% critical speed, the shoulder position becomes high, the steel balls projected into flight follow a parabolic path, and much higher number of steel balls follow a cataracting motion. As the mill speed increases from 90% to 100% critical speed, the steel balls are almost projected flight follow a centrifuging motion, large number of steel ball has a high speed collide on the lifter and result in the wear rate of lifter increases.

DEM software provides an accurate prediction of the steel ball trajectory in the ball mill. However, the above discussion is dependent on observing the steel ball trajectory in photographs. Simply observing the trajectory is not enough to describe what is occurring inside the ball mill. Hence, an impact force sensor was mounted in the lifter to investigate impact load behavior.

#### 4.2.2. Effect of Mill Speed on Impact Force

**Figure 6:**
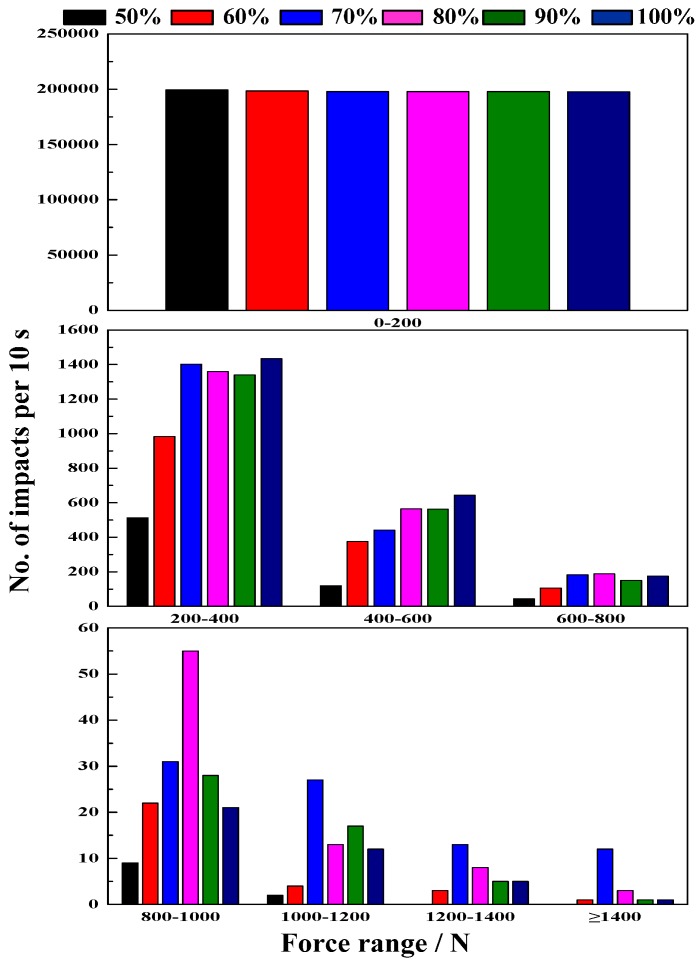
Impact force at different mill speeds.

Figure 6 shows the results of impact force at different mill speeds. Increasing the mill speed can increase the impact load behavior, but an excessive increase in mill speed results in higher energy consumption. As shown in Figure 6, the frequency of impacts in a force range of 0–200 N is the maximum at lower speeds. In lower speeds of 50–60% critical speed, the impact force is obviously weak, a large number of steel ball move at a lower speed and has lower impact energy, resulting in particle abrasive comminution. At 70–80% critical speed, a force range of 600–1400 N has the maximum percent compared with other mill speeds. Hence, the lifter has a higher impact force and results in more crushing and grinding. Additionally, at 90–100% critical speed, the steel ball move at a high speed and increasing the rate of the lifter, but forces ranging primarily from 0 to 600 N results in more grinding.

From the above results, we can conclude that mill speed has a significant influence on load behavior. The impact force of the lifter for investigating multi-body load behavior is a useful method. At lower speeds (50–60% of critical speed), steel balls from the toe position to shoulder position cascade down along the charge surface, so the load behavior is mainly in a grinding process. At higher speeds (70–80% of critical speed), the steel balls are projected to fall down and impact the toe position, so the load behavior is mainly in a crushing process. However, an excessive speed result in steel balls impacting space position, and increases the lifter wear.

#### 4.2.3. Effect of Mill Speed on Power Draw

**Figure 7:**
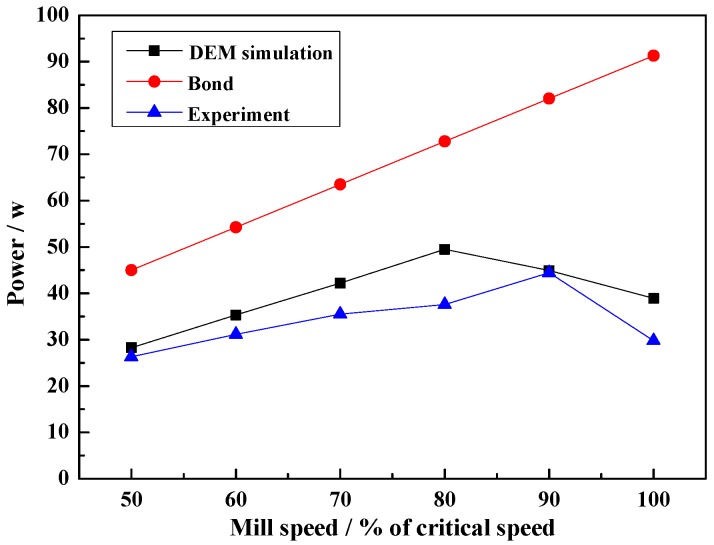
Power draws at different mill speeds.

Figure 7 shows the results of mill speed on power draw. The DEM simulation and experimental power draw increased and then decreased with mill speed, the results of the DEM simulation agree well with the experiment results. However, the Bond semi-empirical power draw model increases with mill speed, and the power draw value is larger than in the experiment and DEM simulation by approximately 1.71–3.07 times. This result shows that the accuracy of Bond formula is the lowest when the mill speeds at 100% of critical speed.

In the DEM simulation and experiment, the power draw increases with mill speed up to a peak value of 80% of critical speed. This means that the optimum grinding efficiency occurs at mill speeds of near 80% of critical speed [23], because more steel balls are projected into the cataracting region. After 80% of critical speed, the mill power draw decreases until 100% of critical speed and more steel balls begin centrifuging, leading to a power draw decrease to drive the mill. This outcome demonstrates that the DEM simulation is an accurate calculation method. In the Bond formula, this result indicates that Bond formula is not suitable for predict the power accurately in this condition, and the major difference can be explained by the fact that Bond formula does not consider the effect of the lifters. In addition, Bond formula applied data collected from industrial mills and laboratory tests results, the model based on both torque-arm or energy balance principals and some coefficients, and the calculate results are affected by the mill length and diameter, ball density, ball filling and rotation speed. 

From the above results and discussions, the mill speed crucially affects the charge motion, impact force and power draws. In order to obtain an efficient grinding, the mill speed between 70% and 80% can be beneficial to ball mill operation. Furthermore, the ball mill with one transparent end and a photographed trajectory of charge can offer accurate input parameters for DEM simulation. 

### 4.3. Effect of Grinding Media Filling on Impact Load Behavior

#### 4.3.1. Steel Ball Trajectories of DEM Simulation

**Figure 8:**
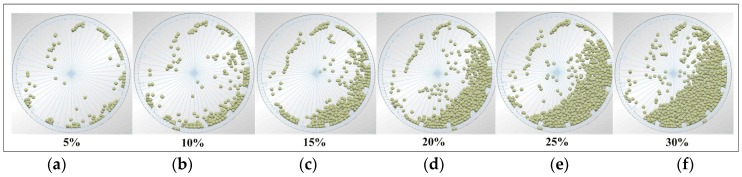
Photograph of the impact load behavior with 75% critical speed at 5–30% grinding media filling: (**a**) 5%; (**b**) 10%; (**c**) 15%; (**d**) 20%; (**e**) 25%; and (**f**) 30%.

Figure 8 shows the steel ball trajectories on different grinding media filling, with the filling changed from 5% to 30%, a lifter size of 40 × 20 × 20 mm, and a mill speed of 75% [23] critical speed. Increasing the grinding media filling not only increases the fraction flying in the cataracting zone but also increases the rate of ball on ball contact per unit. Commonly, increasing the filling level increases the impact energy, and effective impact increases.

#### 4.3.2. Effect of Grinding Media Filling on Impact Force

**Figure 9:**
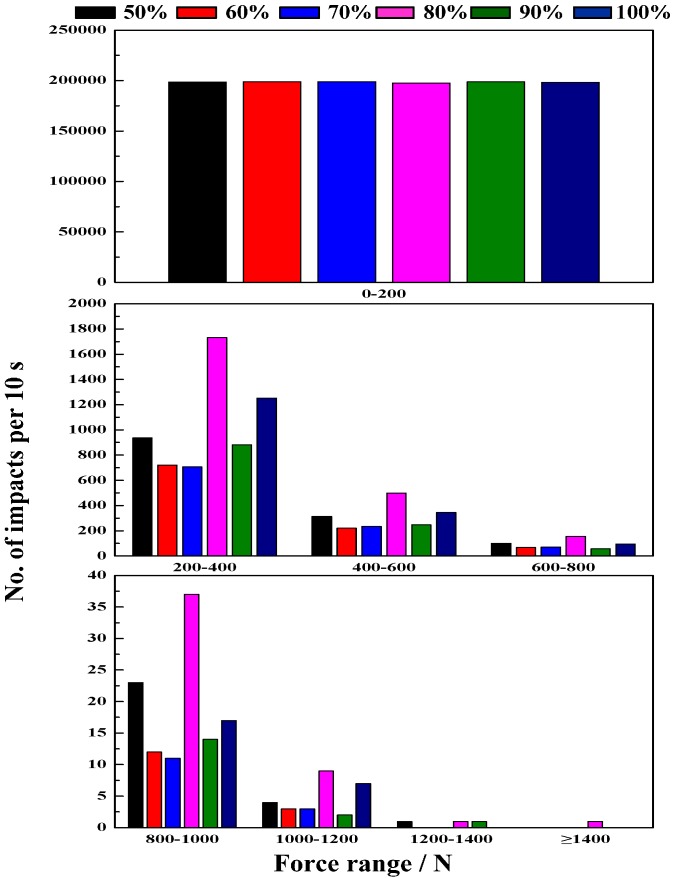
Impact force at different grinding media filling.

Increasing the filling not only increases the impact load but also results in more wearing and breakage of the lifter. Hence, a suitable filling level is critical to improving efficiency. Figure 9 shows the impact force on the lifter at different filling levels. The impact force can be only above 1400 N at the ball filling of 20%, and the maximum percent of impact force between 200 and 1400 N is obtained at the grinding media filling of 20%. Increasing the filling level results in more ball to ball collisions, but the frequency of steel ball direct impacts on the lifter does not always increase.

From the above investigation, increasing the grinding media filling does not increase the impact force on the lifter. Due to the lifter profile and rotation speed at the same conditions, the number of impacts on the lifter does not change when the filling level up to a suitable level. Therefore, further increases the filling level results in more steel balls impacting the space region and energy consumption.

#### 4.3.3. Effect of Grinding Media Filling on Power Draw

**Figure 10:**
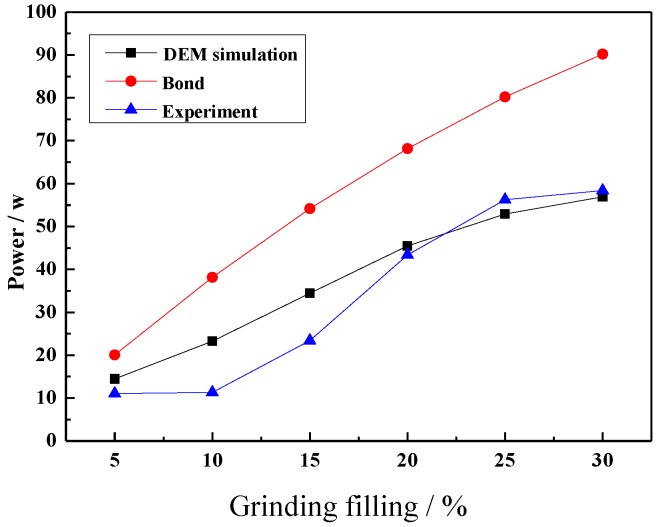
Power draw at different grinding media filling.

Figure 10 shows the results of power draw at different grinding media fillings. This shows that increasing the filling level leads to an increase in the power draw. As the filling level increases, the variation between the Bond semi-empirical power draw and experiment increases. The Bond semi-empirical power draw model is 1.51–1.82 times greater than the experimental result. Additionally, the experiment power draw value at 5–10% and 25–30% grinding media filling does not significantly change, and the DEM simulation result has a larger difference at 10% and 15% grinding media filling.

From the above results, this Bond formula is only suitable for a rough estimation of the mill power draw, and the Bond semi-empirical power draw model is less accurate than the DEM simulation. For the Bond semi-empirical power model, the main difference is that the calculation result does not consider the effect of the lifter. For the DEM simulation, this difference can be explained by the effect of the contact parameter. Still increasing the grinding media filling could not increase the milling efficiency according the impact force on the lifter. As a result, a suitable grinding media filling has a beneficial to ball mill grinding process. 

### 4.4. Effect of Lifter Height on Impact Load Behavior

#### 4.4.1. Steel Ball Trajectories of DEM Simulation

**Figure 11:**
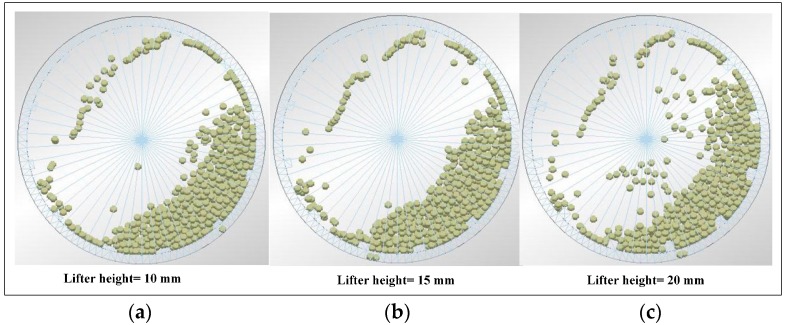
Photograph of the impact load behavior with different lifter height: (**a**) 10 mm; (**b**) 15 mm; and (**c**) 20 mm.

Figure 11 shows the steel ball trajectories at different lifter heights, grinding media filling of 20%, and a mill speed of 75% critical speed. Increasing the lifter height obviously increases the number of cataracting steel balls. 

In the milling process, due to wear and impact, the lifter height gradually decreases until it is too low and must be replaced. This condition causes the steel ball not to reach a sufficient location, and the number of high–speed steel balls decreases. Finally, if the lifter height is too low, the working efficiency of the ball mill decreases. Therefore, the lifter height is a significant factor influences the steel ball trajectories.

#### 4.4.2. Effect of Lifter Height on Impact Force

**Figure 12:**
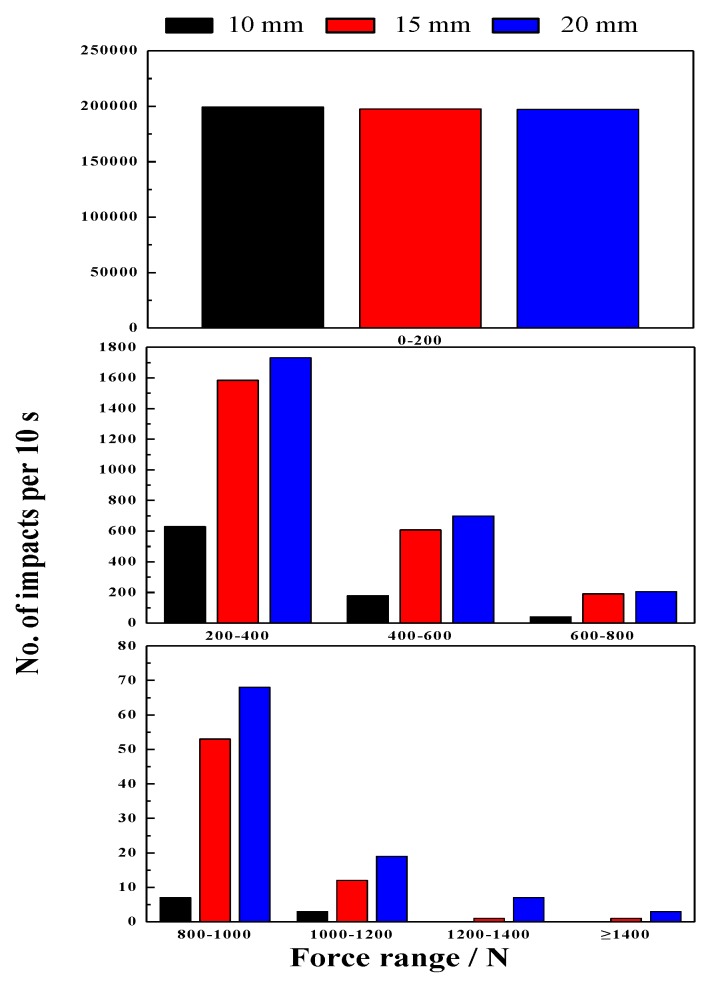
Impact force at different lifter heights.

Figure 12 shows the result of impact force on the lifter for different lifter heights. Increasing the lifter height lifts the steel balls to an increased height and results in higher impact velocity. As the lifter height increases from 10 to 20 mm, the steel balls contact the mill shell earlier and impact force increases. Increasing the lifter height increases the shoulder position and lifts the steel balls to increased heights the higher position. The percent of impact force ranging from 0 to 200 N decreases with the increase of lifter height. However, this perfect will increase above 200 N. 

From the above results, it can be concluded that lifter height has a significant influence on working efficiency. Since the steel balls are lifted much higher when the lifter height increases, many high-speed steel balls impact the lifter. Therefore, effectively changing the lifter height helps to improve the milling efficiency.

#### 4.4.3. Effect of Lifter Height on Power Draw

**Figure 13:**
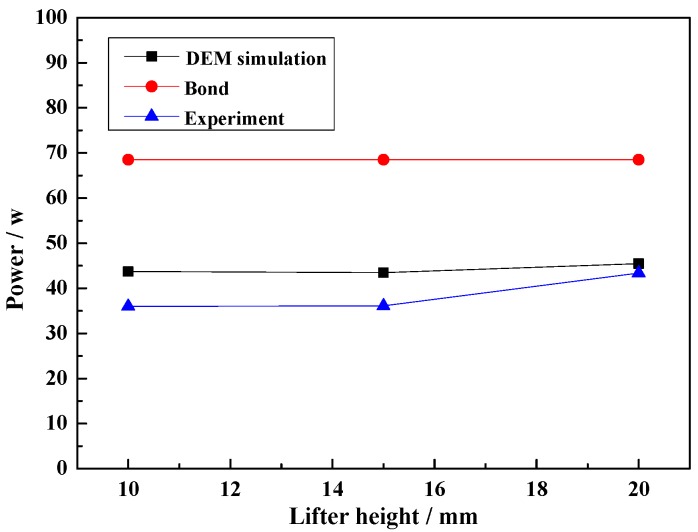
Power draw at different lifter heights.

Figure 13 shows the effect of lifter height on power draw. Increasing the lifter height has a less significant effect on power draw. For the Bond semi-empirical power draw model, the power draw values are still much greater than the experimental values by approximately 1.58–1.9 times. For the DEM simulation, the power draw values are greater than the experimental values by approximately 1.05–1.21 times.

From the above results, the Bond semi-empirical power draw model is only suitable for estimating the power draw, and difference is influenced by the lifter. Besides, the difference between experiments and DEM simulation results are determined by the distance between the center of the mill and the center of gravity of the load. Thus, the DEM simulation can predict the load behavior at different lifter heights when the input parameters are accurate.

From the above results and discussions, the lifter height has a significant effect on charge motion and impact force, but little effect on power draw. Increasing the lifter height increases the lifting ability, and the lifter height is related to the charge diameter. Thus, it is important to choose a suitable lifter height for a determined ball mill. 

### 4.5. Effect of Media-Shape on Impact Load Behavior

#### 4.5.1. Effect of Particle on Load Behavior

**Figure 14:**
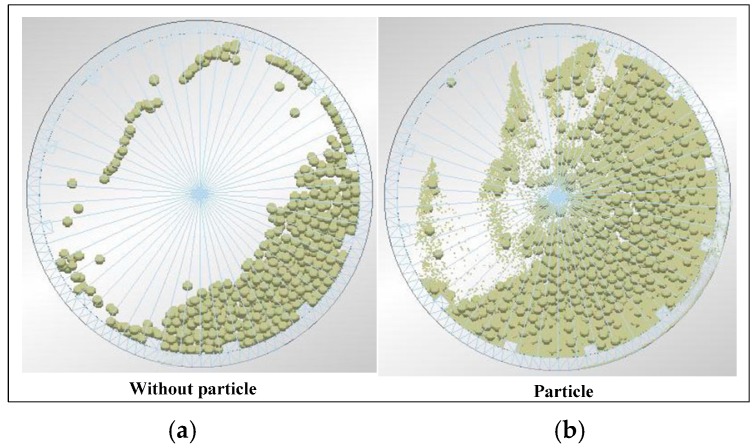
Photograph of the impact load behavior: (**a**) without particle and (**b**) with particle.

Figure 14 shows the effect of particle on load behavior. The mill has a lifter size of 40 × 20 × 15 mm, mill speed of 75% critical speed, grinding media filling of 20%, and powder grinding media ratio of 0.8. Adding iron ore particles increases the height of charge, and more charges are projected into the cataracting zone. 

#### 4.5.2. Effect of Particle on Impact Force

**Figure 15:**
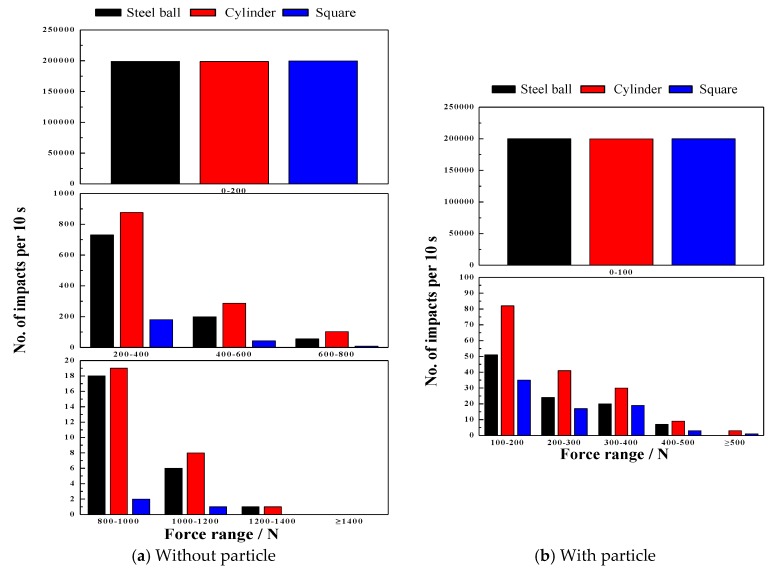
Effect of particle on impact force. (**a**) Without particle; (**b**) With particle.

The charge, the particle and lifter which collide together form a complex environment in the mill shell. To investigate the real grinding process, a simulation was performed to obtain the influence of particles and different media shapes on the impact breakage behavior. The results are shown in Figure 15. Adding iron ore particles significantly reduced the impact force from 1400 to 500 N. From Figure 15a,b, the number of impacts for a force range of 0–200 N in order are square, steel ball and cylinder shaped charge and for other force ranges are cylinder, steel ball and square shaped charge.

From the above results, it can be determined that media shape has a significant influence on the impact force in the lifter. The results occur because of the media shape of the steel ball, cylinder and square is point, line and surface contact, respectively. Moreover, adding iron ore particles buffers the impact force on the lifter, which decreases the impact force. 

#### 4.5.3. Effect of Particle on Power Draw

**Figure 16:**
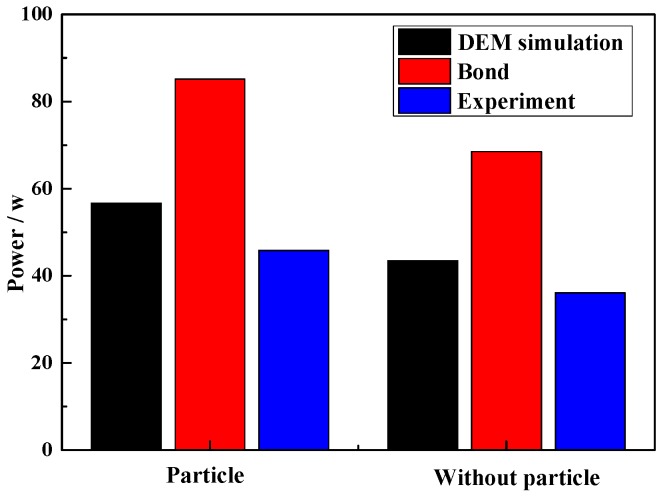
Power draw with and without particles.

To further compare the effect of adding iron ore particles on the grinding process, the mill power and particle size distribution are investigated. As shown in Figure 16, adding iron ore particles increases the power draw, and results in a power draw increase from 36.10 increases to 45.82 W at experimental measurement. Due to the iron ore increases the mill filling, so the power draw increases. For the Bond predict result, the DEM simulation results is more accurately.

**Figure 17:**
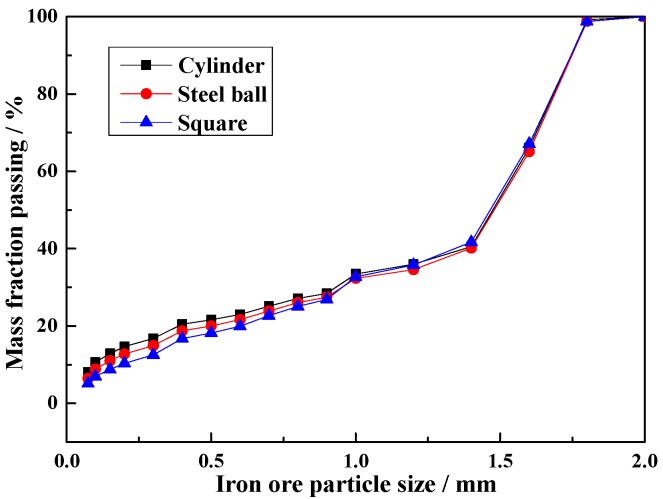
Iron ore particle size distribution.

To study the difference of media shape on the milling process, an iron ore particle size distribution analysis is the most direct way to explain the effect of multi-body impact breakage behavior of different media shapes. As shown in Figure 17, the mass fraction passing of cylinder is greater than that of ball and square. The percent of −0.074 mm particle is 8.08% (cylinder), 6.42% (ball) and 5.14% (square), respectively. The cylinder, steel ball and square shapes have line, point and surface contact, so the cylinder projected to impact the particle has the optimum effect. This result also demonstrates the effect of different media shapes on the impact force in the lifter.

From the above results and discussions, it can be concluded that different media shapes have a different influence on grinding efficiency. Besides, adding the particle in the ball mill can influence the milling process. Therefore, choosing a suitable media shape based on the milling environment is an effective method for improving grinding efficiency.

## 5. Conclusions

In this paper, a laboratory-scale ball mill is employed in investigating the impact of different operating variables on the impact behavior of lifter. The determinations of ball trajectory, impact force and power draw are used to characterize the impact behavior between the charge and lifter. The main research conclusions are summarized as follows.

- The magnitude of impact force has strong sensitive to ball size and drop height. The impact force increases with the increase of ball size and drop height.
- Mill speed has profound effect on the ball trajectories, impact force and power draw. The mill can reach the best performance at the mill speed ranging from 70% to 80% of critical speed, and correspondingly the maximum percent of the impact force between 600 and 1400 N is obtained. For the 80% of critical speed, the measured power draw has a maximum value.
- Commonly, increasing the grinding media filling not only increases the fraction flying in the cataracting zone but also increases the rate of ball on ball contact per unit. In this research, it is interesting to find that increasing the grinding media filling does not the number of ball on ball contact. The impact force can be only above 1400 N at the grinding media filling of 20%, and the maximum percent of impact force between 200 and 1400 N is obtained at the grinding media filling of 20%. Besides, increasing the grinding media filling increases the power draw.
- Lifter height has a significantly influence on the load behavior. Increasing the lifter height increases the shoulder position and lifts the steel balls to the higher position,. The percent of impact force ranging from 0 to 200 N decreases with the increase of lifter height. However, this perfect will increase above 200 N. In general, increasing the lifter height does not lead to any variation in power draw.
- The impact force will decrease and the power draw will increase when the iron ore particles are added. The mass fraction passing of cylinder is greater than that of ball and square. The percent of −0.074 mm particle is 8.08% (cylinder), 6.42% (ball) and 5.14% (square), respectively.
- The semi-empirical power draw of Bond model in this paper does not apply very satisfactorily for the ball mills, while the power draw determined by DEM simulation show a good approximation for the measured power draw.

## Figures and Tables

**Table 1:** Experimental conditions.

Test	Mill Speed/of Critical Speed	Filling Level	Lifter Height (mm)	Media-Shape	Particle
4.2	50%, 60%, 70%, 80%, 90%, 100%	20%	20	Steel ball	None
4.3	75%	5%, 10%, 15%, 20%, 25%, 30%	20	Steel ball	None
4.4	75%	20%	10, 15, 20	Steel ball	None
4.5	75%	20%	15	Steel ball, Square, Cylinder	None
4.5	75%	20%	15	Steel ball, Square, Cylinder	Yes

**Table 2:** Material properties.

Materials	Size (L = Length; W = Width; H = Height)		
Grinding media	Steel ball (∅15 mm)	Square (L: 12 × W: 12 × H: 12 mm)	Cylinder (∅12 × H: 16 mm)
Iron ore particle	2–2.8 mm		
Lifter	L: 40 × W: 20 × H: 10 mm	L: 40 × W: 20 × H: 15 mm	L: 40 × W: 20 × H: 20 mm

**Table 3:** The parameters of DEM simulations.

Material Parameters	Value
Iron ore particle density (kg/m^3^)	3886
Iron ore particle shear modulus (Pa)	2.59 × 10^9^
Iron ore particle Poisson’s ratio	0.28
Shell density (kg/m^3^)	7800
Shell shear modulus (Pa)	7 × 10^10^
Shell Poisson’s ratio	0.3
Particle-shell restitution coefficient	0.32
Particle-shell static friction coefficient	0.48
Particle-shell rolling friction coefficient	0.2
Particle-Particle restitution coefficient	0.49
Particle-Particle static friction coefficient	0.48
Particle-Particle rolling friction coefficient	0.16
